# Inhibition of Mutation and Combating the Evolution of Antibiotic Resistance

**DOI:** 10.1371/journal.pbio.0030176

**Published:** 2005-05-10

**Authors:** Ryan T Cirz, Jodie K Chin, David R Andes, Valérie de Crécy-Lagard, William A Craig, Floyd E Romesberg

**Affiliations:** **1**Department of Chemistry, The Scripps Research InstituteLa Jolla, CaliforniaUnited States of America; **2**The Department of Medicine, Section of Infectious DiseaseUniversity of Wisconsin Medical School, Madison, WisconsinUnited States of America; **3**Molecular Biology, The Scripps Research InstituteLa Jolla, CaliforniaUnited States of America; Tufts University School of MedicineUnited States of America

## Abstract

The emergence of drug-resistant bacteria poses a serious threat to human health. In the case of several antibiotics, including those of the quinolone and rifamycin classes, bacteria rapidly acquire resistance through mutation of chromosomal genes during therapy. In this work, we show that preventing induction of the SOS response by interfering with the activity of the protease LexA renders pathogenic Escherichia coli unable to evolve resistance in vivo to ciprofloxacin or rifampicin, important quinolone and rifamycin antibiotics. We show in vitro that LexA cleavage is induced during RecBC-mediated repair of ciprofloxacin-mediated DNA damage and that this results in the derepression of the SOS-regulated polymerases Pol II, Pol IV and Pol V, which collaborate to induce resistance-conferring mutations. Our findings indicate that the inhibition of mutation could serve as a novel therapeutic strategy to combat the evolution of antibiotic resistance.

## Abstract 2

The evolution of bacterial resistance to antibiotics presents a serious public-health threat, but may be inevitable because mutation is stimulated by exposure to some antibiotics. Inhibition of mutational mechanisms should slow resistance.

## Introduction

The worldwide emergence of antibiotic-resistant bacteria threatens to undo the dramatic advances in human health that were ushered in with the discovery of these drugs in the mid-1900s. Today, resistance has rendered most of the original antibiotics obsolete for many infections, mandating an increased reliance on synthetic drugs. However, bacteria also evolve resistance to these drugs, typically by acquiring chromosomal mutations [1–6]. Within the classical paradigm that mutations are the inevitable consequence of replicating a large genome with polymerases of finite fidelity, resistance-conferring mutations are unavoidable. However, recent evidence suggests that bacteria may play a more active role in the mutation of their own genomes in response to at least some DNA-damaging agents by inducing proteins that actually promote mutation [7–15]. If the acquisition of antibiotic resistance-conferring mutations also requires the induction of these proteins, then their inhibition would represent a novel approach to combating the growing problem of drug resistance.

In an initial effort to examine the role of induced mutation in the evolution of antibiotic resistance, we have focused our studies on the synthetic antibiotic ciprofloxacin. Ciprofloxacin is a member of the quinolone family of antibiotics, which is rapidly becoming the most important family of antibiotics [16]. Quinolones function by interfering with the two essential type II DNA topoisomerases in bacteria, gyrase and topoisomerase IV [17]. These topoisomerases normally function by forming a protein-bridged DNA double strand break (DSB), manipulating DNA strand topology, and finally rejoining the DNA ends. Ciprofloxacin reversibly binds to the protein-bridged DSB intermediate and inhibits rejoining of the DNA ends. The toxic effects of ciprofloxacin may be the result of topoisomerase subunit dissociation without re-ligation of the DNA ends [17,18], likely producing free double strand ends (DSEs) when the protein-DNA bond is eventually hydrolyzed or the DNA is processed by a nuclease. In addition, covalently bound topoisomerases may also block DNA replication forks, which after processing will also produce DSEs [17,19–23]. Resistance to ciprofloxacin requires mutations in the genes that encode the topoisomerases (*gyrA* and *gyrB*, encoding gyrase, and *parC* and *parE*, encoding topoisomerase IV) or in the genes that affect cell permeability or drug export [1,17].

To understand how mutation could be induced by ciprofloxacin, it is essential to consider how bacteria respond to the presence of the antibiotic. Previously it was suggested that ciprofloxacin-mediated DNA damage may be repaired by nucleotide excision repair (NER) or homologous recombination (HR) [17]. RecA-single-stranded DNA (ssDNA) filaments play an important role in these processes by catalyzing strand invasion of a DNA end into a homologous sequence. However, RecA-ssDNA filaments also play an important role in induced mutation by binding the SOS [10] gene repressor LexA and unmasking its autoproteolytic activity [24]. Upon autoproteolysis, LexA no longer represses the approximately 30 genes whose protein products facilitate the repair of DNA damage, delays in cell cycle division, and mutation. In particular, sufficient reduction of the cellular concentration of LexA repressor results in the transcription of the genes encoding Pol II *(polB),* Pol IV *(dinB),* and Pol V *(umuD* and *umuC),* which are three nonessential DNA polymerases that have been shown to be required for mutation in response to DNA damage [10,25–28].

Because ciprofloxacin may induce repair pathways that involve RecA-ssDNA filament formation, the drug itself may act to induce the mutations that confer resistance. This hypothesis is consistent with observations that quinolones can be mutagenic and can induce the SOS response [17]. Here, using an in vivo infection model, we show that interfering with LexA autoproteolysis renders pathogenic Escherichia coli unable to mutate and acquire resistance to ciprofloxacin. The same result is demonstrated in vivo for the antibiotic rifampicin, which is a semisynthetic member of the rifamycin class of antibiotics that inhibits the bacterial RNA polymerase. The in vivo results are recapitulated in vitro for ciprofloxacin (here, we use in vitro to refer to bacteria in liquid cultures or on solid media). To understand how LexA cleavage is induced by ciprofloxacin and how this cleavage mediates resistance, we examined the contribution of a variety of different genes to ciprofloxacin tolerance and resistance in vitro. We show that E. coli repairs ciprofloxacin-induced DNA damage primarily by RecBC-dependent HR, and to a lesser extent by illegitimate recombination (IR). Finally, we show that Pol II, Pol IV, and Pol V are all required for the evolution of resistance.

## Results

### LexA Cleavage Is Required for the Evolution of Resistance to Ciprofloxacin and Rifampicin In Vivo

**Figure 1:**
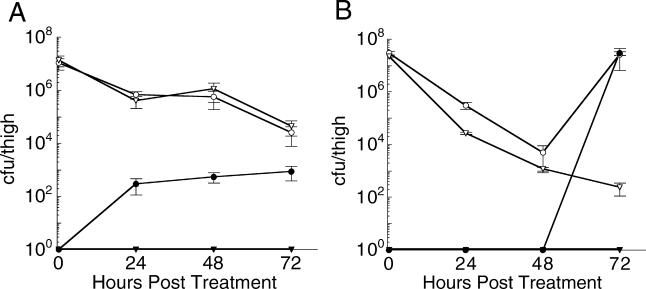
Survival and Mutation of E. coli Mutants In Vivo after Starting Antibiotic Therapy Survival and mutation of Δ*lacZ* and *lexA*(S119A) mutants of E. coli ATCC 25922 in thighs of neutropenic mice at 24-h intervals after starting therapy with (A) ciprofoxacin or (B) rifampicin. Open circles and triangles correspond to the total cfu/thigh of the Δ*lacZ* and *lexA*(S119A) strains, respectively. Solid circles and triangles represent the number of drug-resistant Δ*lacZ* and *lexA*(S119A) cfu/thigh, respectively.

To test whether activation of the SOS response is required to induce the mutations that confer ciprofloxacin resistance during therapy, we used a neutropenic murine thigh infection model [29] and the pathogenic E. coli strain ATCC 25922. Mice were infected with 10^6^ colony-forming units (cfu) of either the Δ*lacZ* (control) or *lexA*(S119A) 25922 strain (Table 1). The LexA(S119A) mutant cannot undergo autoproteolysis and thus renders bacteria unable to de-repress the SOS genes [30]. After infection, ciprofloxacin (0.5 mg/kg) was administered every 12 h. After 24, 48, and 72 h, mice were sacrificed, thigh tissue was homogenized, and dilutions of the homogenate were plated on media with and without ciprofloxacin to quantify the number of viable and resistant cfu, respectively (Figure 1A). Treatment with ciprofloxacin had the same, slow bactericidal effect on both strains, reducing the total cfu/thigh approximately 250-fold over the 72 h of treatment. In mice infected with the Δ*lacZ* control strain, significant resistance emerged, amounting to approximately 3% of the entire population by 72 h. Individual clones were found to have ciprofloxacin minimum inhibitory concentrations (MICs) as high as 640 ng/ml (ten clones analyzed), significantly increased from the 12 ng/ml MIC observed for the Δ*lacZ* strain before infection. In dramatic contrast, no resistant mutants were isolated from mice infected with the *lexA*(S119A) strain. The MICs of the *lexA*(S119A) clones recovered from the mouse thigh and isolated from the ciprofloxacin-free media remained approximately equal to that of the strain prior to infection (approximately 12 ng/ml, ten clones analyzed).

**Table 1:**
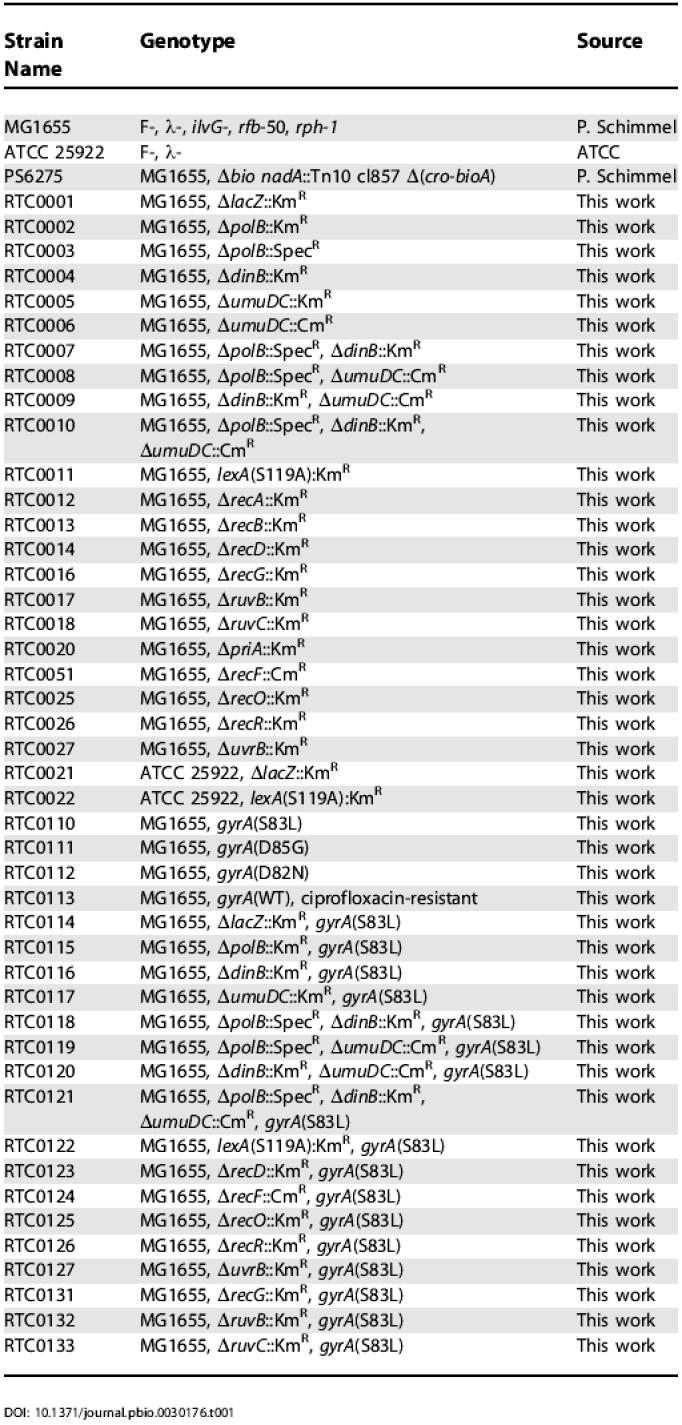
Strains Used in This Work

We next used the same mouse model to characterize the evolution of resistance to rifampicin in vivo. Again, mice were infected with 10^6^ cfu of either the Δ*lacZ* control or the *lexA*(S119A) 25922 strain. Rifampicin (100 mg/kg) was administered every 12 h, mice were sacrificed after 24, 48, and 72 h, and the total number of viable and rifampicin-resistant cfu was determined. Treatment with rifampicin also had a slow bactericidal effect on both strains (Figure 1B); however, by 72 h all of the Δ*lacZ* clones recovered from the thigh were resistant to rifampicin. MICs of individual rifampicin-resistant Δ*lacZ* clones were all higher than 250 μg/ml (ten clones analyzed), which is substantially higher than the 8 μg/ml MIC of the parent strain. Remarkably, despite the rapid acquisition of resistance in the control strain, no resistant clones were isolated from mice infected with the *lexA*(S119A) strain. The MICs of the *lexA*(S119A) clones recovered from the mouse thigh remained 8 μg/ml, approximately the same as the parent strain (ten clones analyzed). We conclude that LexA cleavage is absolutely required for the evolution of resistance to both ciprofloxacin and rifampicin during therapy in vivo.

### Ciprofloxacin Induces Biochemical Pathways That Facilitate Mutation In Vitro

**Figure 2:**
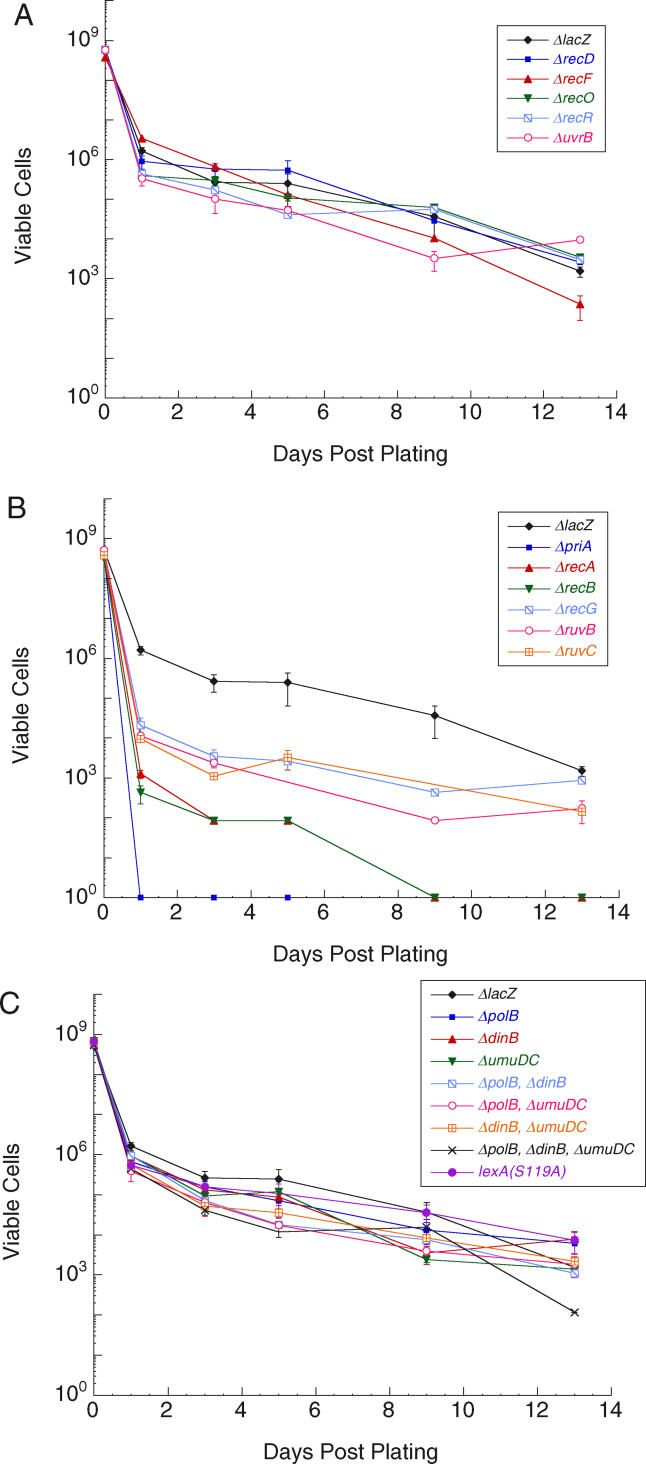
Survival of E. coli Mutants In Vitro Survival on solid media containing 40 ng/ml ciprofloxacin of Δ*lacZ* control and (A) NER and recombination mutants with wild-type sensitivity, (B) recombination mutants that were hypersensitive to ciprofloxacin, and (C) *lexA*(S119A) and inducible polymerase mutants.

In order to further characterize the genetic requirements of these resistance-conferring mutations, we studied bacteria in vitro. We constructed an isogenic series of mutants in the E. coli strain MG1655 (MG1655 is not pathogenic, but it has been fully sequenced [31], which facilitated the construction of the required deletion strains) (Table 1). As with the in vivo studies, a Δ*lacZ* strain was constructed as a control (the Δ*lacZ* strain exhibited wild-type growth and mutation). We first determined that the ciprofloxacin MIC for the Δ*lacZ* control strain is 35 ng/ml in liquid media (Table 2). On solid media, we found that 40 ng/ml ciprofloxacin killed 99% of the cells within 24 h of plating, while the remaining 1% of the population persisted for several weeks, allowing for the characterization of the bacteria in the presence of the antibiotic (Figure 2).

**Table 2:**
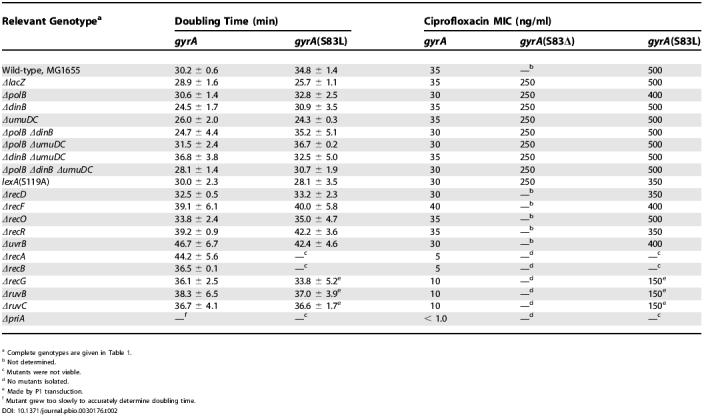
Growth and Ciprofloxacin Sensitivity of MG1655-Derived Strains

^a^ Complete genotypes are given in Table 1.

^b^ Not determined.

^c^ Mutants were not viable.

^d^ No mutants isolated.

^e^ Made by P1 transduction.

^f^ Mutant grew too slowly to accurately determine doubling time.

We measured the rate at which the Δ*lacZ* control strain evolves resistance in vitro on solid media containing 40 ng/ml ciprofloxacin; at this concentration, single mutations (typically in *gyrA*) are sufficient to confer resistance (Tables 2 and S1) [1]. Δ*lacZ* cells were grown in permissive liquid culture and then plated onto ciprofloxacin-containing media. Resistant colonies were counted as they arose, in 24 h intervals over 14 d. Colonies that formed immediately (at or before day 2) were attributed to cells that had acquired resistance prior to exposure to ciprofloxacin (via “pre-exposure” mutations), while colonies that formed on day 3 or later were attributed to cells that acquired resistance after exposure to ciprofloxacin (via “post-exposure” mutations). Assignments of pre- and post-exposure mutations were validated by using two different reconstruction assays designed to confirm when the mutations occurred (see Materials and Methods). Mutation rates were defined as the number of resistant colonies that arose per time, per viable cell (it should be noted that this experiment detects only those mutations that confer resistance). We observed a pre-exposure mutation rate of 9.0 (± 9.5) × 10^−10^ mutants/viable cell/d, and a post-exposure mutation rate of 1.8 (± 0.69) × 10^−5^ mutants/viable cell/d (Table 3). Thus, ciprofloxacin induces resistance by a factor of 10^4^. These rates are in agreement with those reported previously by Hall using a similar in vitro assay [32].

**Table 3:**
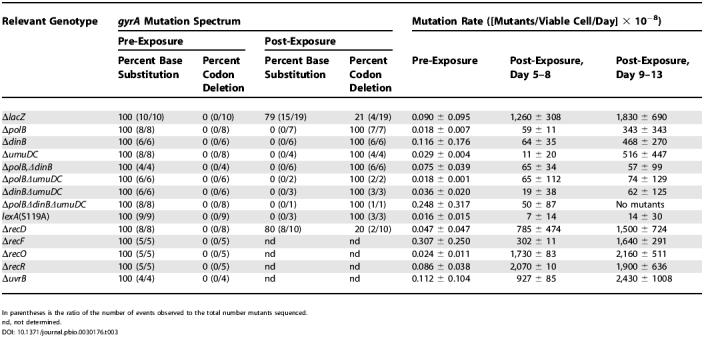
Mutation Spectra and Rates

In parentheses is the ratio of the number of events observed to the total number mutants sequenced.

nd, not determined.

We next examined the mutation spectrum of the *gyrA* gene in the resistant clones by sequencing a 1,000-nt region encompassing the quinolone resistance-determining region [33]. We found that the spectrum of the post-exposure mutations differed significantly from the pre-exposure mutations (Table 3). In the pre-exposure mutants (ten clones sequenced) we observed strictly substitution mutations, while in the post-exposure mutants (19 clones sequenced) we observed both substitutions and a three-basepair, in-frame deletion that removed the codon for Ser83. This “codon deletion” occurred despite a lack of flanking direct repeats or palindromic sequences, which are often thought to facilitate spontaneous deletions.

### LexA Cleavage Is Required for the Acquisition of Resistance In Vitro

To characterize the role of LexA cleavage and the SOS response in the evolution of resistance in vitro, we constructed a *lexA*(S119A) MG1655 strain. Mutation of LexA did not affect growth or persistence in the presence of 40 ng/ml ciprofloxacin (Figure 2C and Table 2). Previously, another noncleavable LexA mutant, *lexA*(G85D), was shown to be moderately sensitive to high levels of ciprofloxacin (6× MIC) [34,35]. The difference in sensitivities associated with these two noncleavable LexA mutants is likely the result of different levels of ciprofloxacin-mediated DNA damage, i.e., an increased reliance on the induction of SOS genes under the more damaging conditions employed in the *lexA*(G85D) study.

We observed the same in vitro pre-exposure mutation rate and spectrum for the *lexA*(S119A) strain as we did for the control strain (Table 3). However, the *lexA*(S119A) strain exhibited a post-exposure mutation rate that was approximately 100-fold lower than that observed for the Δ*lacZ* control strain (Table 3). The decreased evolution of resistance did not depend on the concentration of the antibiotic, as virtually identical results were obtained with 60 ng/ml of ciprofloxacin (unpublished data). Given the low mutation rate, only three post-exposure ciprofloxacin-resistant *lexA*(S119A) mutants were isolated (from more than 10^11^ bacteria plated, overall), but all three acquired resistance by deletion of the Ser83 codon and not by substitution mutation (Table 3). Similar results were observed in vitro with the Δ*lacZ* and *lexA*(S119A) 25922 strains used in the in vivo studies, both in terms of mutation rates (Figure S1) and mutation spectrum. To ensure that the reduced ability to evolve resistance observed with the *lexA*(S119A) strain was not simply because these cells grew slowly with gyrase mutations, we examined the *lexA*(S119A) *gyrA* double mutants (Figure S2 and Table 2). Relative to the respective control strain, each double mutant showed virtually identical growth in the absence of ciprofloxacin, virtually identical persistence in the presence of ciprofloxacin, and identical ciprofloxacin MICs. In all, the data indicate that the decreased evolution of resistance in the *lexA* mutant did not result from decreased persistence or the inability to grow upon acquisition of a *gyr* mutation, but rather from an inability to induce mutations.

The evolution of clinically significant resistance requires the stepwise accumulation of several mutations [1]. To test whether LexA cleavage is required for these additional mutations, we examined the in vitro evolution of resistance to 650 ng/ml ciprofloxacin in Δ*lacZ* control and *lexA*(S119A) MG1655 strains already containing the prototypical “first step” Ser83Leu mutation in *gyrA* that confers resistance to 40 ng/ml ciprofloxacin (strains RTC0114 and RTC0122; see Table 1). The “second step” mutation rate was 1.9 (± 0.21) × 10^−4^ mutants/viable cell/d in the control strain and 5.5 (± 4.9) × 10^−7^ mutants/viable cell/d in the *lexA*(S119A) strain (Figure S3). Assuming that the first and second step mutations are independent, the LexA mutant strain evolves resistance to 650 ng/ml ciprofloxacin in vitro with a rate that is approximately 10^4^-fold lower than the control strain. Because clinical resistance typically requires three to five independent mutations [1], the data imply that in the absence of efficient LexA cleavage, E. coli would evolve clinical resistance at least 10^6^-fold slower. These in vitro results fully recapitulate the in vivo mouse model studies and demonstrate that LexA cleavage-mediated derepression of one or more genes is essential for the efficient evolution of resistance.

### RecBC-Mediated Homologous Recombination Likely Induces LexA Cleavage in the Presence of Ciprofloxacin

To understand how the presence of ciprofloxacin leads to LexA cleavage, we characterized a series of MG1655 strains harboring deletions of genes involved in various DNA repair pathways (Tables 1 and and S2). We reasoned that a pathway important for mediating ciprofloxacin-induced LexA cleavage would involve the formation of RecA-ssDNA filaments and also would be induced by the drug. Because the ciprofloxacin-topoisomerase complexes act to cross-link DNA, repair may occur by NER, mediated by UvrA, UvrB, and UvrC, followed by recombinational gap repair, mediated by RecF, RecO, RecR, and RecA [36–39]. We tested whether this classical cross-link repair pathway is induced by the antibiotic by examining the sensitivity and the pre- and post-exposure mutation rates for Δ*uvrB,* Δ*recF,* Δ*recO,* and Δ*recR* strains. No differences in sensitivity (see Figure 2A and Table 2) or mutation rate (Table 3) were observed compared to the control strain, suggesting that NER and RecFOR-mediated recombinational gap repair are not induced in the presence of ciprofloxacin or required for the efficient evolution of resistance. A previous study of *uvrB* and *recF* deletion strains suggested that they were more sensitive to nalidixic acid than was the wild-type strain [37]. However, unlike the current study, the previous study involved short incubations at high drug concentrations. Thus, the different experimental conditions are likely responsible for the different results.

Because topoisomerase dissociation from the DNA may generate free DSBs, repair may occur by a combination of HR and DNA replication in a process generally referred to as recombination-dependent DNA replication (RDR) [40,41]. In *E. coli,* RDR is mediated by RecA filamentation on ssDNA, created by the helicase/nuclease RecBCD, and PriA-dependent replisome assembly. To test whether the RecBCD pathway of HR is induced in the presence of ciprofloxacin, we examined the Δ*recA,* Δ*recB,* and Δ*recD* strains. Although the Δ*recA* and Δ*recB* strains exhibited virtually wild-type viability, both were hypersensitive to ciprofloxacin (see Figure 2B and Table 2). No pre- or post-exposure mutants were observed in either strain. To investigate whether resistant colonies were not isolated because the *gyrA*(S83L) Δ*recA* and *gyrA*(S83L) Δ*recB* double mutants are not viable, we attempted to construct these strains by P1 transduction of the *rec* deletion into a *gyrA*(S83L) strain. No viable double mutants could be constructed in either case. This is consistent with previous reports that certain mutant gyrase proteins cause increased spontaneous fork collapse, which makes the cells dependent on HR [42–45]. Thus, we propose that RecA/RecBC-mediated recombination is important for cell viability in the presence of ciprofloxacin both before and after the acquisition of resistance-conferring mutations. In contrast, deletion of *recD* had no effect on drug sensitivity (see Figure 2A and Table 2), mutation rate, or mutation spectrum (Table 3). This result is consistent with the fact that RecBC can process DSEs and load RecA onto ssDNA in the absence of the RecD helicase [46].

Because persistent covalently-linked topoisomerase-DNA complexes will eventually block the progression of replication forks, repair may occur by recombination-dependent fork repair, which is a variant of RDR that reestablishes a processive replication fork [22,41,47–50]. In addition to the proteins that mediate HR (i.e., RecA and RecBC) this process appears to require RecG and RuvABC. To test whether recombination-dependent fork repair is induced in the presence of ciprofloxacin, we examined the Δ*recG,* Δ*ruvB,* and Δ*ruvC* strains. Deletion of *recG, ruvB,* or *ruvC* did not cause a significant decrease in viability in the absence of ciprofloxacin, but did result in high sensitivity to the drug, although not as high as that observed with deletion of *recA* and *recB* (see Figure 2B and Table 2). Like the Δ*recA* and Δ*recB* strains, no pre- or post-exposure mutants were isolated. However, we were able to delete *recG, ruvB,* and *ruvC* in a *gyrA*(S83L) strain by P1 transduction. These double mutants grew as well as the corresponding single mutants (see Table 2). While the double mutants each displayed significantly reduced MICs relative to the *gyrA*(S83L) single mutant (see Table 2), they were still able to form colonies on media containing 40 ng/ml ciprofloxacin. Thus, selection against resistance-conferring mutants in the *recG, ruvB,* and *ruvC* backgrounds does not explain the absence of resistant colonies. These results suggest that the functions of RecG, RuvB, and RuvC are required for viability in the presence of ciprofloxacin (although less so than RecA and RecB) and also for the acquisition of resistance-conferring mutations.

Because PriA-dependent reinitiation of DNA synthesis is important for a variety of HR models [49,51,52], it may be required for the repair of ciprofloxacin-mediated DNA damage. Deletion of *priA* resulted in extreme sensitivity to ciprofloxacin (MIC of less than 1 ng/ml), implying that replication restart is essential in response to the drug (see Figure 2B and Table 2). As with the *rec* and *ruv* strains, no mutants were isolated before or after exposure to ciprofloxacin, and as with the Δ*recA* and Δ*recB* strains, we were unable to construct the *gyrA*(S83L) Δ*priA* double mutant. These results imply that replication restart is essential in response to ciprofloxacin and in tolerating the resistance-conferring gyrase mutations.

### Induction of the LexA-Repressed Polymerases Does Not Contribute to Survival but Is Critical for the Evolution of Ciprofloxacin Resistance In Vitro

To characterize how LexA cleavage induces resistance, we examined the role of the three LexA-repressed polymerases, Pol II *(polB),* Pol IV *(dinB),* and Pol V *(umuD* and *umuC)* in survival and mutation in vitro. In every respect, the effects of deleting a single LexA-repressed polymerase (i.e., Δ*polB,* Δ*dinB,* or Δ*umuDC*), or any combination of the three, were the same as the effects of preventing LexA cleavage. First, the *pol* deletion strains showed similar sensitivities to ciprofloxacin as the control strain (Figure 2C and Table 2). (The Δ*polB,* Δ*polB ΔdinB,* Δ*polB ΔumuDC,* and Δ*polB ΔdinB ΔumuDC* strains were all slightly sensitive to ciprofloxacin, which is consistent with a role for replication restart in the response to the drug, as replication restart is thought to involve Pol II [1,41,53]). Second, the MIC of each *gyrA pol* mutant was virtually identical to that of the corresponding *gyrA* single mutant (Table 2). Third, the pre-exposure mutation rate and spectrum of the *pol* deletion strains were the same as the control; however, the post-exposure rates were markedly reduced (Table 3). Finally, resistance in the post-exposure mutants was acquired strictly through deletion of either Ser83 or Ala84, and not through substitution (29 clones sequenced; Table 3). These results suggest that it is through the derepression of all three repressed polymerases that LexA cleavage induces substitution mutations.

## Discussion

The discovery and development of antibiotics revolutionized medicine, providing easy cures for previously untreatable diseases. However, for every significant infectious disease caused by bacteria, strains resistant to all available antibiotics have been reported [54]. We are interested in understanding how bacteria evolve resistance, and have first focused on the antibiotic ciprofloxacin. Ciprofloxacin and the other quinolones are perhaps the most important antibiotics currently available [16], partly because of the low levels of resistance currently observed with these newer synthetic drugs. However, clinical resistance to the quinolones is evolving at an alarming rate due to mutations in gyrase, topoisomerase IV, and efflux pumps or their regulators [1]. In this study we have shown, in vivo, that preventing LexA cleavage renders bacteria unable to evolve resistance to either ciprofloxacin or rifampicin in a mouse thigh infection model. In vitro, the ability of bacteria to induce mutation and evolve resistance to ciprofloxacin is also dramatically reduced by rendering LexA uncleavable. Thus, our results indicate that the mutations that confer resistance to ciprofloxacin and rifampicin are not simply the result of unavoidable errors accumulated during genome replication, but rather are induced via the derepression of genes whose protein products act to significantly increase mutation rates.

In principle, part of the observed increase in mutation rate after exposure to ciprofloxacin could result from selection against resistant mutants during the pre-exposure growth in liquid media (thus underestimating the pre-exposure mutation rates). However, the impact of selection is unlikely to be significant, as *gyrA* mutations are tolerated without a significant increase in doubling time (Table 2). Further evidence that the resistance-conferring mutations are induced is provided by the fact that deletion or mutation of several genes, including *lexA,* renders cells unable to evolve significant levels of resistance.

**Figure 3:**
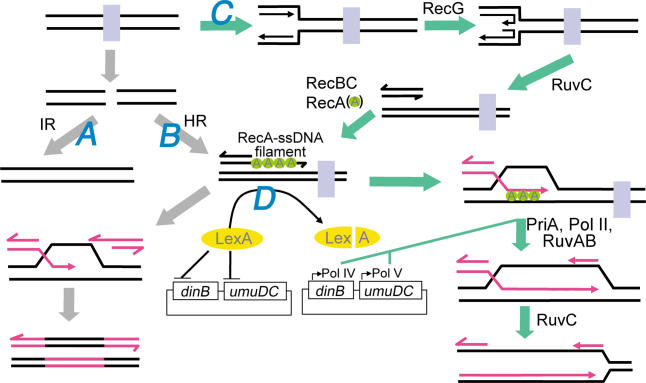
Proposed Response to Ciprofloxacin In the absence of homologous sequences, free DSBs are repaired by nuclease and polymerase-dependent IR (pathway A). In the presence of a suitable homologous sequence, free DSBs may be repaired by RDR (pathway B). This involves resectioning of the DNA ends by RecBC and loading of RecA onto the ssDNA produced. These RecA-ssDNA filaments catalyze D-loop formation and repair of the DSB. This pathway may also contribute to the repair of replication forks when they encounter the free DSB. Finally, replication forks that encounter topoisomerases that are covalently-bound to the DNA are repaired by recombination-dependent fork repair (pathway C). This involves RecG-mediated fork regression and RuvC cleavage to produce DSEs where RecBC mediates RecA-ssDNA filament formation. These filaments catalyze strand invasion of a homologous sequence where PriA, and possibly Pol II, help to reestablish a processive replication fork. With sufficient accumulation of DSBs and collapsed forks, persistent RecA-ssDNA filaments induce levels of LexA cleavage sufficient to de-repress the error prone polymerases, Pol IV and Pol V, which cooperate to induce mutations (pathway D). Once resistance-conferring mutations are made, DSBs and collapsed forks cease to accumulate and RecA-filaments no longer persist. Subsequently, the cellular concentration of LexA increases, shutting down expression of the pro-mutagenic polymerases. See text for details.

The data suggest that the increase in mutation rate is caused by recombination pathways that are induced to repair antibiotic-mediated DNA damage. A mechanism consistent with our results is illustrated in Figure 3. Three recombinational repair pathways appear to be involved that are distinguished by the type of damage they repair and the type of mutation they induce. One pathway is IR-mediated repair of free DSBs where the protein has dissociated from the DNA (pathway A, Figure 3). We suggest that this pathway is responsible for the observed codon deletions. The induction of small nucleotide deletions has been observed in bacteria [55–57], and in eukaryotes, it has been suggested that small deletions (including codon deletions) arise during IR after an inhibited topoisomerase aberrantly releases free DSBs, the ends of which are processed by exonucleases and polymerases before being rejoined [58]. While we may have observed a similar phenomenon in our in vitro studies, the codon deletion mutants are unlikely to be of much clinical significance on their own, as they have relatively low ciprofloxacin MICs (Table 2) and have never been observed in the clinic [1,33]. Isolation of these mutants in the current study was most likely due to the permissive drug concentrations employed.

In addition to IR, RDR and replication fork repair are also induced to repair DSBs in cases where the topoisomerase has dissociated from, or remains bound to, the DNA, respectively (pathways B and C, Figure 3). These pathways may be more relevant to clinical resistance as they induce the substitution mutations ubiquitously found in clinically resistant strains. In both pathways, the RecBCD nuclease/helicase loads at DSEs generated (directly or indirectly) by ciprofloxacin and simultaneously degrades and unwinds the duplex while loading RecA onto the ssDNA of the nascent 3′-overhang. (For replication fork repair, RecG and RuvABC are required to prepare the DSE [50,59,60].) RecA forms filaments that promote strand invasion of the ssDNA into a homologous sequence, resulting in the formation of an intermediate known as a displacement-loop structure (D-loop). The invading strand may then prime DNA synthesis, using the homologous sequence as a template, ultimately restoring the genetic information disrupted by the DSE [48]. In the case of replication fork repair, the covalently bound topoisomerase must still be displaced from DNA in order to reinitiate processive synthesis. We propose that the topoisomerase is displaced by RuvAB, which has recently been shown to branch migrate D-loop-like structures and simultaneously displace covalently-bound ciprofloxacin-topoisomerase IV complexes [61], or perhaps by Rep or UvrD helicase, which have both been shown to displace bound proteins from duplex DNA [62]. Following PriA recognition and binding, a processive replication fork is reestablished. However, with the continued presence of ciprofloxacin these processes will continue, resulting in the persistence of the RecA-ssDNA filaments, which eventually degrade enough LexA to derepress the error-prone, SOS-regulated polymerases (pathway D, Figure 3).

The data suggest that the induction of substitution mutations requires the derepression of all three SOS-regulated polymerases, Pol II, Pol IV, and Pol V. While the evolution of resistance to ciprofloxacin by substitution mutation is to our knowledge the first process found to require all three of the E. coli inducible polymerases, this observation is consistent with previous studies showing that multiple polymerases are required for some mutations [9,41,63,64]. It is also consistent with the two-step model of translesion synthesis, wherein one specialized polymerase is required for dNTP misinsertion and another for continued synthesis (mispair extension) [9,27,65]. We propose that the induced mutations conferring antibiotic resistance in vitro and in vivo are the result of Pol V mispair synthesis [66,67] and Pol IV mispair extension [10,68], while Pol II may be required to initiate replication restart [41, 53] (after which it may be replaced by Pol V and then Pol IV), or to fix the nascent mutation by extending the primer terminus sufficiently to avoid exonucleolytic proofreading upon reloading of Pol III [69]. This process continues until mutations are made that allow for the resumption of normal DNA synthesis.

The key signal that links the cellular response to the antibiotic with the evolution of resistance appears to be the RecA-ssDNA filaments that are formed to facilitate the repair of antibiotic-mediated DNA damage. These RecA-ssDNA filaments also induce LexA cleavage and derepression of the mutagenic polymerases. We suggest that a similar mechanism might also serve to induce mutation and evolution in response to other antibiotics, or other forms of cellular stress, where DNA damage per se is not involved. For example, the ratio of ATP to ADP determines the level of supercoiling in the bacterial genome [17], and both increased and decreased levels of supercoiling inhibit replication fork progression [70]. Thus, different stresses that perturb metabolism (i.e., alter ATP/ADP ratios) might also alter DNA topology and result in stalled replisomes; recombination-based rescue and RecA-ssDNA filament formation; and the induction of mutations required to reestablish a normal cellular environment. Interestingly, it has recently been shown that β-lactams can induce the SOS response via a two-component signal transduction system [71].

The traditional paradigms of DNA replication and mutation suggest that resistance-conferring mutations are the inevitable consequence of polymerase errors, and offer no obvious means for intervention. In stark contrast, the model described above suggests that bacteria play an active role in the mutation of their own genomes by inducing the production of proteins that facilitate mutation, including Pol IV and Pol V, as has been suggested with other forms of mutation [7–15]. In turn, this suggests that inhibition of these proteins, or the prevention of their derepression by inhibition of LexA cleavage, with suitably designed drugs, might represent a fundamentally new approach to combating the emerging threat of antibiotic-resistant bacteria. Future efforts will focus on determining the generality of the observations, in terms of both other pathogenic bacteria and other antibiotics.

## Materials and Methods

### 

#### Bacterial strains and growth**

Strains of E. coli used in this study are listed in Table 1. Solid media was Lennox LB [72] plus 1.6% agar; liquid media was Miller LB [72]. For selection in MG1655- and ATCC 25922-derived strains, antibiotics were used as follows: kanamycin, 30 and 50 μg/ml; spectinomycin, 100 μg/ml; and chloramphenicol, 20 μg/ml. All bacteria were grown at 37 °C unless otherwise indicated. Ciprofloxacin and rifampicin were purchased from MP Biomedicals (Aurora, Ohio, United States). For strain construction and additional experimental details, see Protocol S1.

A standard mouse infection model was employed [29]. Briefly, 6-wk-old, specific-pathogen-free, female CD-1 mice (weight, 23–27 g; Harlan Sprague Dawley) were used. Mice were rendered neutropenic (neutrophil counts less than 100/mm^3^) by intraperitoneal injection with 150 mg/kg cylcophosphamide (Mead Johnson Pharmaceuticals, Evansville, Indiana, United States) 4 d before infection and 100 mg/kg cyclophosphamide 24 h before infection. Previous studies have shown that this regimen produces neutropenia in this model for 5 d [73]. Mueller-Hinton (MH) (Difco) broth cultures inoculated from freshly plated bacteria were grown to logarithmic phase (OD_580_ of approximately 0.3), and diluted 1:10 in MH broth. Thigh infections were produced by injecting 0.1 ml volumes (approximately 10^6^ cfu) of the diluted broth cultures into halothane-anesthetized mice. Starting 2 h after infection (defined as time zero), mice were administered subcutaneous injections of either 0.5 mg/kg ciprofloxacin or 100 mg/kg rifampicin every 12 h for 3 d. After 24, 48, and 72 h, both thighs from two sacrificed animals were removed and homogenized. Serial dilutions of homogenates of each thigh were plated on MH agar (MHA) and MHA containing either 80 ng/ml ciprofloxacin or 128 μg/ml rifampicin (lower limit of detection was 100 organisms/thigh). MICs for both ciprofloxacin and rifampicin were determined by standard microdilution methods of the National Committee for Clinical Laboratory Standards. MICs prior to drug exposure for both the LexA mutant and control strains were determined by examining ten clones isolated from MHA plates. MICs of the post-exposure isolates from both the LexA mutant and control strains were determined by examining ten clones isolated from the MHA plates at the 72-h time point.

#### Mutation assay.

For each strain, five independent cultures were grown for 25 h without ciprofloxacin. Viable cell counts in these cultures were determined by plating serial dilutions onto permissive media. For assaying mutation in MG1655-derived strains, 150 μl from each culture (approximately 10^8^ cells) was plated in duplicate on LB/agar containing 40 ng/ml ciprofloxacin. Five additional 150 μl aliquots from two cultures of each strain were also plated on the same media for use in the “survival” assay (see below). ATCC 25922-derived strains were assayed similarly with 12 ng/ml ciprofloxacin, except cultures were concentrated approximately 3-fold before plating to assay the same number of cells as in the MG1655 experiments. At 24 h intervals, visible colonies were counted, their location on the plate was marked, and they were stocked at −80 °C for later use in the reconstruction assay (see below).

#### Survival assay.

Every 24 h, in parallel with the mutation assay, all visible colonies were excised from plates designated for assaying survival (see above), the remaining agar was homogenized in saline, and dilutions were plated in duplicate on LB/agar to determine the total number of viable, ciprofloxacin-sensitive cells present as a function of time, and LB/agar containing 40 ng/ml ciprofloxacin to determine if any ciprofloxacin-resistant colonies remained after excision. An experimental validation of this method is described in Protocol S1 and Table S3.

#### Reconstruction assay.

We determined whether colonies isolated after plating on ciprofloxacin formed as a result of post-exposure mutation or as a result of mutation prior to exposure to the drug. Liquid cultures of permissive media were inoculated with ciprofloxacin-resistant clones stocked during the mutation assay (see above) and grown to saturation overnight. Cultures were diluted and plated in duplicate on both LB/agar, to confirm viability, and LB/agar containing 40 ng/ml ciprofloxacin, to confirm resistance. Clones that were resistant before exposure were defined as those that formed colonies on the ciprofloxacin-containing media in the same number of days in the reconstruction assay as they did in the original mutation assay. Conversely, clones that mutated after exposure to ciprofloxacin were defined as those that formed colonies at least 2 d faster in the reconstruction assay.

To further confirm our assignment of the pre- and post-exposure mutants, an alternative method was also employed. Ciprofloxacin-resistant clones isolated during the mutation assay were suspended in 1 ml of 9 mg/ml NaCl. A series of dilutions was plated onto permissive media (LB/agar) and plates were incubated at 37 °C for 24–48 h. From each set of dilution plates, a plate was chosen that contained approximately 50–300 colonies. This plate was replica-plated onto permissive and nonpermissive media (LB/agar with 40 ng/ml ciprofloxacin), and replica plates were incubated at 37 °C. Replica plates were analyzed at 24 h intervals for the appearance of colonies (Figure S4). At the same time, the ciprofloxacin-resistant clones were also assayed using the reconstruction method described above. The two reconstruction assays gave identical results.

#### Calculation of the rate at which cells become resistant to ciprofloxacin (mutation rate).

Mutation rate was defined as the number of ciprofloxacin-resistant mutants per viable cell that evolve as a function of time. We emphasize that it reflects only those mutations that both allow cells to survive and confer resistance to the drug. The mutation rate before exposure to ciprofloxacin (pre-exposure rate) was determined by fluctuation analysis and application of the *p_0_* method [74]. The mutations after exposure to ciprofloxacin (post-exposure rate) exhibited the expected Poisson distribution [7–15] and the associated rate was therefore determined as the ratio of colonies on a particular day to the number of cells present at the time the cells became resistant, which we approximated as the viable cell count 2 d prior. The assumption that a colony takes 2 d to form accounts for both the actual time required for colony growth and the time required to turn over any residual ciprofloxacin-sensitive protein, i.e., phenotypic lag. After determining the post-exposure mutation rate for each day from days 3–13, rates were averaged over days 3–4, 5–8, and 9–13. Error bars reflect standard deviation in rate determinations from at least three independent sets of experiments.

## Supporting Information

Figure S1 In Vitro Mutation Rate of ATCC 25922-Derived Strains
Mutation rate of ATCC 25922 (1), ATCC 25922-Δ*lacZ* (2), and ATCC 25922-*lexA*(S119A) (3). Bars represent total mutation rate (base substitution and codon deletion).
(59 KB TIF).

Figure S2 *gyrA* Mutant Growth on Ciprofloxacin Is Not Affected by Genetic Background Growth of *gyrA*(S83L) mutant clones in the (A) Δ*lacZ*, (B) Δ*polB* Δ*dinB* Δ*umuDC*, and (C) *lexA*(S119A) genetic backgrounds on LB/agar containing 40 ng/ml ciprofloxacin. Plates were photographed after incubation at 37 °C for 24 h.
(945 KB TIF).

Figure S3 Stepwise Mutation Rate Stepwise mutation rate of Δ*lacZ*, *gyrA*(S83L) (1) and *lexA*(S119A), *gyrA*(S83L) (2) to 650 ng/ml ciprofloxacin.
(48 KB TIF).

Figure S4 Reconstruction of Mutants by Replica Plating Re-growth of eight Δ*lacZ* ciprofloxacin-resistant colonies isolated during the mutation assay on (A) Day 5, (B and C) Day 6, (D and E) Day 7, (F) Day 8, (G) Day 9, and (H) Day 10, 48 h after replica-plating onto LB/agar (left) and LB/agar containing 40 ng/ml ciprofloxacin (right). This reconstruction method demonstrates that all of the cells from a colony isolated during the mutation assay are ciprofloxacin resistant. See Materials and Methods for details.
(2.4 MB TIF).

Protocol S1 Supplementary Methods (195 KB DOC).

Table S1 Effect of Different Ciprofloxacin-Resistance Mutations (28 KB DOC).

Table S2 Primers Used in This Work (72 KB DOC).

Table S3 Validation of Survival Assay (42 KB DOC).

### Accession Numbers

The TIGR (http://www.tigr.org) Locus Names for the genes discussed in this paper are *dinB* (NT01EC0267), *gyrA* (NT01EC2662), *lacZ* (NT01EC0408), *lexA*(NT01EC4829), *polB* (NT01EC0071), *priA* (NT01EC4700), *recA* (NT01EC3205), *recB* (NT01EC3353), *recD* (NT01EC3352), *recF* (NT01EC4430), *recG* (NT01EC4373), *recO* (NT01EC3056), *recR* (NT01EC0568), *ruvB* (NT01EC2229), *ruvC* (NT01EC2233), *umuC* (NT01EC1406), *umuD* (NT01EC1405), and *uvrB* (NT01EC0934).

## Abbreviations

cfu: colony-forming units
DSB: double strand break
DSE: double strand end
HR: homologous recombination
IR: illegitimate recombination
MIC: minimum inhibitory concentration
NER: nucleotide excision and repair
RDR: recombination-dependent replication
ssDNA: single-stranded DNA
