# Supplementary material for: Inhibition of Mutation and Combating the Evolution of Antibiotic Resistance
Source: PLoS Biol. 2005 May 10;3(6):e176. doi: 10.1371/journal.pbio.0030176 (PMC1088971; doi:10.1371/journal.pbio.0030176)
Supplement: Supplementary file 1 [file pbio.0030176.sd001.doc]

**Protocol S1.**

Inhibition of mutation and combating the evolution of antibiotic resistance

*Ryan T. Cirz, Jodie K. Chin, David R. Andes, Valérie de Crécy-Lagard,*

*William A. Craig and Floyd E. Romesberg*

**Strain Construction**. Strains were constructed by first generating a disruption cassette using 3-way PCR as described by Murphy [1]. Deletion cassettes consisted of approximately 500 bp regions upstream and downstream of the gene to be deleted, including the first and last 2-10 codons of the gene, flanking an antibiotic resistance cassette in reverse orientation. *E. coli* specific sequences were amplified from MG1655 genomic DNA and purified with the DNeasy Tissue kit (Qiagen). The Km^R^ cassette was amplified from pUC4K [2], the Spec^R^ cassette from pOmega [3], and the Cm^R^ cassette from pSU18 [4]. Oligonucleotide primers used in the construction of the disruption cassettes are listed in Table S1.

The *lexA*(S119A) mutation cassette was constructed by a similar method. Approximately 500 bp of sequence upstream of the *lexA* coding sequence and the complete coding region of *lexA* was PCR-amplified from genomic MG1655 DNA using the primers lexA_NF-SphI and lexA_OrfR-NdeI, digested with *Sph*I/*Nde*I and ligated into *Sph*I/*Nde*I digested pUC18 vector [5]. The S119A mutation (TCG–>GCG) was introduced in the resulting plasmid using the Quikchange Site-directed Mutagenesis kit (Stratagene) with the primers LexA_S119A_QCF and LexA_S119A_QCR. The resulting allele was confirmed by sequencing. A 500 bp fragment corresponding to the genomic sequence downstream of *lexA* was amplified with the primers lexA_CF and lexA_CR. The Km^R^ cassette was amplified from pUC4K using the primers KanF and KanR. The 500 bp downstream fragment was PCR assembled with the Kan cassette using the 20 bp homology intrinsic in primer lexA_CF (See Table S1). The pUC18 vector containing 500 bp of upstream DNA and the *lexA*(S119A) coding sequence was digested with *Sph*I/*Nde*I and the Kan cassette/downstream fragment assembly product was digested with *Nde*I. The two products were ligated together and PCR amplified using the primers lexA_NF-SphI and lexA_CR. The final *lexA* cassette consisted of 500 bp of upstream DNA, the mutated *lexA* ORF, an *Nde*I site attaching a Km^R^ marker in reverse orientation, and approximately 500 bp of downstream DNA (see below).

*lexA*(S119A)*:*Km^R^ cassette

The disruption cassettes were transformed by electroporation into strain PS6275, plated on LB supplemented with the appropriate antibiotic, and grown at 30 °C. After confirmation of correct chromosomal insertion by PCR, cassettes were transferred into MG1655 by P1 transduction [6] and confirmed by plating on minimal media lacking biotin and containing the antibiotic used for cassette selection. Correct disruption was confirmed by PCR; the *lexA*(S119A) strain was confirmed by PCR followed by sequencing.

To construct the ATCC 25922 derivatives of the ∆*lacZ* and *lexA*(S119A) mutants, the respective cassettes were transferred by P1 transduction from strains RTC0001 and RTC0011 into ATCC 25922; colonies were selected on LB supplemented with 50 μg/ml kanamycin. Transfer of the mutant alleles was confirmed by PCR followed by sequencing for the *lexA*(S119A) mutant.

The *gyrA* mutant clones from different genetic backgrounds described in Table 2 and Table S2 were isolated during mutation assays. Because no *gyrA* mutant clones could be isolated in the *∆recG, ∆ruvB,* and *∆ruvC* backgrounds, the three knockout cassettes were each moved by P1 transduction into RTC0110 to create strains RTC0131, RTC0132, and RTC0133, respectively (see Table 1 and Table 2).

#### Growth Rate Determination. For each strain, two independent cultures were grown for 25 h at 37 °C in LB containing no antibiotic. These cultures were diluted 1:500 in LB and grown at 37 °C with shaking. At each time point, the A_600_ was measured and cultures were diluted and plated on LB/agar to determine the number of viable cells. Growth from 90-300 was used to determine the doubling times for each strain (Table 2).

#### Minimum Inhibitory Concentration Determination. For each strain, two independent cultures were grown for 25 h at 37 °C in LB containing no antibiotic. From each culture, ~10^4^–10^5^ colony forming units (cfu) were used to inoculate, in duplicate (total of 4 data points per strain), LB containing 12 different concentrations (0, 1.0, 3.0, 5.0, 7.5, 10, 15, 20, 25, 30, 35, 40, 50 ng/ml) of ciprofloxacin using 96-well flat bottom plates. After 18 h of incubation at 37 °C, growth was measured by reading A_650_ in a *Vmax* Kinetic Microplate Reader (Molecular Devices, CA). The MIC was defined as the lowest concentration of ciprofloxacin that prevented any detectable growth. MICs for all strains were determined in at least three independent experiments (total of 12 data points per strain per ciprofloxacin concentration). MICs for ciprofloxacin-resistant clones isolated during the mutation assays were measured with the same conditions used for the non-resistant strains described above, except wells contained LB plus ciprofloxacin at 0, 50, 100, 150, 200, 250, 300, 350, 400, 500, 600, 800, 1000 ng/ml.

##### Sequencing. Colonies from the reconstruction assays were streaked on LB/agar containing 40 ng/ml ciprofloxacin. A single colony from each plate was used as a colony PCR template for *gyrA* gene fragment amplification with the primers gyrA_OrfF and gyrA_1KbR (Table S1). This region of the *gyrA* gene (base pairs 1-1000) contains the quinolone resistance determining region (QRDR) [7], the most consistent location of ciprofloxacin resistance mutations in gram negative bacteria. The PCR products were purified using the Qiaquick PCR Purification kit (Qiagen) and sequenced with the primers gyrA_OrfF and gyrA_Seq (Table S1). The number of mutants sequenced per strain is listed in Table 3. Some ciprofloxacin-resistant clones had no mutations in the *gyrA* region sequenced. MICs of these WT *gyrA* clones were always significantly lower than any clones containing *gyrA* mutations (generally < 80-150 ng/ml, Table S2). These mutant clones most likely contain mutations in the *gyrB*, *parC*, *parE*, *marR*, or *acrR* gene or MarR or AcrR operator sites which could permit growth in the presence of a WT *gyrA* under the conditions used (ciprofloxacin at 40 ng/ml) [8].

#### ‘Second-Step’ Mutation and Survival Assays. A ∆*lacZ*, *gyrA*(S83L) mutant and a *lexA*(S119A), *gyrA*(S83L) mutant were both isolated during mutation assays with 40 ng/ml ciprofloxacin. These mutants were isolated 24 h after plating onto LB/agar plus ciprofloxacin, and thus most likely were resistant prior to plating and exposure to the drug. As a result, these mutants are not likely to have additional second-site mutations. We examined the two clones in mutation, survival, and reconstruction assays as described in the Methods section, with the exception of using media containing 650 ng/ml ciprofloxacin instead of 40 ng/ml.

#### Survival Assay Validation. To validate the accuracy of the survival assay (see Methods), an additional experiment was performed. Three independent cultures of MG1655 were grown overnight at 37 °C. From these cultures, 4 different volumes were plated on minimal media/agar lacking a carbon source (M9 + 1 mM MgSO_4_) and incubated for 1 h at 37 °C. After incubation, plates were homogenized in saline, and the number of cells on each M9 plate was determined (‘Calculated’, Table S3) in a manner analogous to the survival assay. The actual number of cells plated (‘Actual’, Table S3) was determined by plating serial dilutions of the overnight cultures onto LB/agar. In all cases, the calculated number of viable cells on the M9 plates was within ±2-fold of the actual number of cells plated (Table S3).

**Additional Reconstruction Assay**. Our reconstruction assays (see Methods section) were performed in the absence of competing, ciprofloxacin-sensitive cells. Although our mutation assay differs significantly from other systems, the presence of competing cells has affected colony regrowth time in other mutation assays [9]. To address this issue and more accurately re-create the original conditions in which resistant colonies arose in the mutation assay, an additional reconstruction assay was performed for each knockout background/*gyrA* mutant combination isolated during mutation assays. Approximately 100 cfu of the *gyrA* mutant strain were plated on 40 ng/ml ciprofloxacin in the presence of ~10^8^ cells with the same genetic background, but with a wild-type *gyrA* gene. In all cases, colonies from the resistant population arose in the same amount of time as in the reconstruction experiments which lacked competing, ciprofloxacin sensitive cells.

**Growth of *gyrA*(S83L) Mutants in Different Genetic Backgrounds.** For each genetic background that could carry a *gyrA*(S83L) mutation, the ciprofloxacin MIC was measured (Table 2). Strains that have similar MICs should grow similarly on solid media containing ciprofloxacin. However, in order to directly demonstrate this, the ∆*lacZ* *gyrA*(S83L), *lexA*(S119A) *gyrA*(S83L) and ∆*polB* ∆*dinB* ∆*umuDC* *gyrA*(S83L) mutants were each grown overnight in permissive liquid culture and for each strain approximately 100 cfu were plated on LB/agar containing 40 ng/ml ciprofloxacin (the same concentration used in the mutation assay). After incubation for 24 h at 37 °C, the plates all contained substantial, similar sized colonies (Figure S4).

References

1.	Murphy KC, Campellone KG, Poteete AR. (2000) PCR-mediated gene replacement in *Escherchia coli*. Gene 246:321-330.

2.	Muller W, Keppner W, Rasched I. (1986) Versatile kanamycin-resistance cartridges for vector construction in *Escherichia coli*. Gene 46:131-133.

3.	Prentki P, Krisch HM. (1984) In vitro insertional mutagenesis with a selectable DNA fragment. Gene 29:303-313.

4.	Bartolome B, Jubete Y, Martinez E, Cruz F-d-l. (1991) Construction and properties of a family of pACYC184-derived cloning vectors compatible with pBR322 and its derivatives. Gene 102:75-78.

5.	Vieira J, Messing J. (1982) The pUC plasmids, an M13mp7-derived system for insertion mutagenesis and sequencing with synthetic universal primers. Gene 19:259-268.

6.	Miller JH. (1972) Experiments in molecular genetics. Cold Spring Harbor Laboratory Press.

7.	Yoshida H, Bogaki M, Nakamura S, Ubukata K, Konno M. (1990) Nucleotide-sequence and characterization of the *Staphylococcus aureus* *norA* gene, which confers resistance to quinolones. J. Bacteriol. 172:6942-6949.

8.	Lindgren PK, Karlsson Å, Hughes D. (2003) Mutation rate and evolution of fluoroquinolone resistance in *Escherichia coli* isolates from patients with urinary tract infections. Antimicrob. Agents Chemother. 47:3222-3232.

9.	McKenzie GJ, Lombardo M-J, Rosenberg SM. (1998) Recombination-dependent mutation in *Escherichia coli* occurs in stationary phase. Genetics 149:1163-1165.
