# Supplementary material for: Inhibition of Mutation and Combating the Evolution of Antibiotic Resistance
Source: PLoS Biol. 2005 May 10;3(6):e176. doi: 10.1371/journal.pbio.0030176 (PMC1088971; doi:10.1371/journal.pbio.0030176)
Supplement: Supplementary file 2 [file pbio.0030176.st001.doc]

| Table S1. Effect of different ciprofloxacin-resistance mutations | | | |
| --- | --- | --- | --- |
| Strain Name | Genotype | Ciprofloxacin MIC (ng/ml) | Doubling Time  (min) |
| MG1655 | F-, λ-, *ilvG-*, *rfb-*50, *rph-1* | 35 | 30.2 ± 0.6 |
| RTC0110 | MG1655, *gyrA*(S83L) | 500 | 34.8 ± 1.4 |
| RTC0111 | MG1655, *gyrA*(D85G) | 200 | 35.3 ± 1.1 |
| RTC0112 | MG1655, *gyrA*(D82N) | 200 | –^1^ |
| RTC0113 | MG1655, *gyrA*(WT), ciprofloxacin-resistant | 150 | 46.8 ± 2.9 |
| ^1^Not determined. | | | |
