# Supplementary material for: Inhibition of Mutation and Combating the Evolution of Antibiotic Resistance
Source: PLoS Biol. 2005 May 10;3(6):e176. doi: 10.1371/journal.pbio.0030176 (PMC1088971; doi:10.1371/journal.pbio.0030176)
Supplement: Supplementary file 3 [file pbio.0030176.st002.doc]

| Table S2. Primers used in this work | |
| --- | --- |
| Primer Name | Sequence (5'–>3') |
| camF | TCGAGATTTTCAGGAGCTAAG |
| camR | GCCGGCACCAATAACTGCCTT |
| dinB_CF (Kan) | GAGACACAACGTGGCTTTCCCTGGGATTATGATGTATACT |
| dinB_CR | GCTTCGTCATTCGTTCTGCTG |
| dinB_NF | CGCGTGCTGATTAAAGACG |
| dinB_NR (Kan) | GCGGCTTTGTTGAATAAATCGGATTTTACGCATTGCTCACC |
| gyrA_1KbR | CAGGTTCATGATCTTCGGCTG |
| gyrA_OrfF | GATGAGCGACCTTGCGAGAG |
| gyrA_Seq | GTACACCGTCGCGTACTTTAC |
| kanF | GGAAAGCCACGTTGTGTCTC |
| kanR | CGATTTATTCAACAAAGCCGC |
| kanR_NdeI | GGTTGGCATATGCGATTTATTCAACAAAGCCGC |
| lacZ_CF (Kan) | GAGACACAACGTGGCTTTCCGGTCGCTACCATTACCAGTTG |
| lacZ_CR | CAGCCAACACAGCCAAACATC |
| lacZ_NF | CCATGCAAATGCTGAATGAGG |
| lacZ_NR (Kan) | GCGGCTTTGTTGAATAAATCGGGTCATAGCTGTTTCCTGTG |
| lexA_CF | GAGACACAACGTGGCTTTCCCATATCTCTGAGACCGCGATG |
| lexA_CR | GCTTAACCAGCGGATTTCAAG |
| lexA_NF_SphI | TTGGTTGCATGCCAATGGCCAATAATACCACTGG |
| lexA_OrfR_NdeI | GGTTGGCATATGCAGAGATATGTTACAGCCAGTC |
| lexA_S119A_QCF | CCTTCCTTATTCAAGCCGAATGGCGATTTCCTGCTGCGCGTCAGC |
| lexA_S119A_QCR | GCTGACGCGCAGCAGGAAATCGCCATTCGGCTTGAATAAGGAAGG |
| lexA_Seq | CAGGAAGAGGAAGAAGGGTTG |
| polB_CF (Kan) | GAGACACAACGTGGCTTTCCGGGCAACTTGGGCTATTTTGA |
| polB_CFSpec | CTTATGTCCACTGGGTTCGTGGGGCAACTTGGGCTATTTTGA |
| polB_CR | GGTTTGCTGAACACCAGTTTG |
| polB_NF | CATCGAAACCGGTGAAGTGG |
| polB_NR (Kan) | GCGGCTTTGTTGAATAAATCGCTGCGCCACGCTGAAAATCC |
| polB_NRSpec | CTAGCGAGGGCTTTACTAAGCCTGCGCCACGCTGAAAATCC |
| priA_CF (Kan) | GAGACACAACGTGGCTTTCCGGTTAAACCGCTCACGATGCG |
| priA_CR | GATGCGCACAGTCGCCAATCAGC |
| priA_NF | CCGCTGTTTTCGTGGTAATACG |
| priA_NR (Kan) | GCGGCTTTGTTGAATAAATCGGGCAACGGGCATAGCATCATC |
| recA_CF (Kan) | GAGACACAACGTGGCTTTCCGAAGGCGTAGCAGAAACTAAC |
| recA_CR | CGATAGAGCAGAAAACGCTG |
| recA_NF | GTTAAGTGAACAGGTTGGGC |
| recA_NR (Kan) | GCGGCTTTGTTGAATAAATCGGTCGATAGCCATTTTTACTCC |
| recB_CF (Kan) | GAGACACAACGTGGCTTTCCGAGGAGGCGTAATGAAATTGC |
| recB_CR | GGTTTGCGCCAGTAGAGCTTC |
| recB_NF | GCTATGCCGTGTTTGCGTTTC |
| recB_NR (Kan) | GCGGCTTTGTTGAATAAATCGGGCGACATCACTCATTCTTTTCAC |
| recD_CF (Kan) | GAGACACAACGTGGCTTTCCCGGGAATAAACGTAATTGCCG |
| recD_CR | GATTCGTCGCGCAACAATCAACG |
| recD_NF | CGCAATAAACAGGTGGAGATG |
| recD_NR (Kan) | GCGGCTTTGTTGAATAAATCGCAATTTCATTACGCCTCCTCC |
| recF_CF-Cam | CTTAGCTCCTGAAAATCTCGAGATTAACCCAAGTATAAATG |
| recF_CR2 | CCTGCGGTACACCGTGTTCGTAG |
| recF_NF | GCGTAAAGGCGTGATTGAAC |
| recF_NR-Cam | AAGGCAGTTATTGGTGCCGGCGGTGAGGGACATTACAGTCTC |
| recG_CF (Kan) | GAGACACAACGTGGCTTTCCCGTTACTCGAATGCGTAAAAGGCG |
| recG_CR | CGTAAGCTTGTCGTTGTTCC |
| recG_NF | GTTGGCGTACATGAAGTTC |
| recG_NR (Kan) | GCGGCTTTGTTGAATAAATCGGCGACCTTTCATGGCACTTAC |
| recO_CF (Kan) | GAGACACAACGTGGCTTTCCGAATGATGAGGATTGTCATGG |
| recO_CR | GGCGTCAGTTTTGGCATCAGC |
| recO_NF | GCACCTGCAGTTCCTGGCAAG |
| recO_NR (Kan) | GCGGCTTTGTTGAATAAATCGGCCTTCCATCGGAGTTACTC |
| recR_CF (Kan) | GAGACACAACGTGGCTTTCCCATAAGATTCGTTTTTAAGC |
| recR_CR | CTTTGTCGGCCACGATAAACG |
| recR_NF | GGCGTCAGGCGATATACGAAG |
| recR_NR (Kan) | GCGGCTTTGTTGAATAAATCGCATCAGAACGGCATCTTAAAGC |
| ruvB_CF (Kan) | GAGACACAACGTGGCTTTCCGAAATGCCGTAAGTCGGATTG |
| ruvB_CR | CTGTCAATGACGATAAGCCCG |
| ruvB_NF | CACTTTGTGGTGCGTGAAGAC |
| ruvB_NR (Kan) | GCGGCTTTGTTGAATAAATCGGTCTGCTTCAATCATCCTTTACC |
| ruvC_CF (Kan) | GAGACACAACGTGGCTTTCCCGACTGCGTTAAGTTATACCG |
| ruvC_CR | CGTCGGGATCATTATCGGTC |
| ruvC_NF | CCATCATCTACGAAGGTTACG |
| ruvC_NR (Kan) | GCGGCTTTGTTGAATAAATCGGAGAATAATAGCCATCACGC |
| specF | ATTCCCCTGCTCGCGCAGGC |
| specR | GCTTAGTAAAGCCCTCGCTA |
| umuDC_CF (Kan) | GAGACACAACGTGGCTTTCCGTCAAATAAATATAGCGGCAGG |
| umuDC_CFCam | CTTAGCTCCTGAAAATCTCGAGTCAAATAAATATAGCGGCAGG |
| umuDC_CR | CGTGGCTGTTGATGGCGTTTAC |
| umuDC_NF | GTAAGGTTTTAATATCGCCG |
| umuDC_NR (Kan) | GCGGCTTTGTTGAATAAATCGCATAATAATCTGCCTGAAGTTATAC |
| umuDC_NRCam | AAGGCAGTTATTGGTGCCGGCCATAATAATCTGCCTGAAGTTA |
