# Supplementary material for: Inhibition of Mutation and Combating the Evolution of Antibiotic Resistance
Source: PLoS Biol. 2005 May 10;3(6):e176. doi: 10.1371/journal.pbio.0030176 (PMC1088971; doi:10.1371/journal.pbio.0030176)
Supplement: Supplementary file 4 [file pbio.0030176.st003.doc]

## Table S3. Validation of survival assayVolume Plated150 µl100 µl50 µl 15 µlCulture**Actual**CalculatedDeviationActualCalculated**Deviation**ActualCalculatedDeviationActualCalculatedDeviation14.58E+083.13E+081.02E+083.05E+082.56E+083.49E+071.53E+081.04E+083.41E+074.58E+072.81E+071.25E+0725.55E+082.96E+081.83E+083.70E+082.87E+085.86E+071.85E+089.71E+076.21E+075.55E+072.70E+072.02E+0737.35E+083.10E+083.01E+084.90E+082.86E+081.44E+082.45E+081.30E+088.13E+077.35E+073.84E+072.48E+07Average5.83E+083.06E+081.95E+083.88E+082.76E+087.93E+071.94E+081.10E+085.92E+075.83E+073.12E+071.91E+07
